# *Lipoptena fortisetosa* as a vector of *Bartonella* bacteria in Japanese sika deer (*Cervus nippon*)

**DOI:** 10.1186/s13071-021-04585-w

**Published:** 2021-01-22

**Authors:** Shingo Sato, Hidenori Kabeya, Sayuri Ishiguro, Yasuhiro Shibasaki, Soichi Maruyama

**Affiliations:** 1 Laboratory of Veterinary Public Health, Department of Veterinary Medicine, College of Bioresource Sciences, Nihon University, 1866 Kameino, Fujisawa, Kanagawa 252-0880 Japan; 2 Laboratory of Veterinary Food Hygiene, Department of Veterinary Medicine, College of Bioresource Sciences, Nihon University, 1866 Kameino, Fujisawa, Kanagawa 252-0880 Japan; 3 Laboratory of Fish Pathology, Department of Veterinary Medicine, College of Bioresource Sciences, Nihon University, 1866 Kameino, Fujisawa, Kanagawa 252-0880 Japan; 4 Laboratory of Aquatic Animal Health, Department of Marine Science and Resources, College of Bioresource Sciences, Nihon University, 1866 Kameino, Fujisawa, Kanagawa 252-0880 Japan

**Keywords:** *Bartonella*, Deer keds, Ticks, Sika deer, Japan

## Abstract

**Background:**

Two species of deer ked (*Lipoptena cervi* and *L. mazamae*) have been identified as vectors of *Bartonella* bacteria in cervids in Europe and the USA. In an earlier study we showed that Japanese sika deer (*Cervus nippon*) harbor three *Bartonella* species, namely *B. capreoli* (lineage A) and two novel *Bartonella* species (lineages B and C); however, there is currently no information on the vector of *Bartonella* bacteria in sika deer. The aim of this study was to clarify potential vectors of *Bartonella* in Japanese sika deer.

**Methods:**

Thirty-eight wingless deer keds (*L. fortisetosa*) and 36 ticks (*Haemaphysalis* and *Ixodes* species) were collected from sika deer. The prevalence of *Bartonella* in the arthropods was evaluated by real-time PCR targeting the 16S−23S internal transcribed spacer (ITS) and by culture of the organisms. The total number of *Bartonella* bacteria were quantified using real-time PCR. The distribution of *Bartonella* bacteria in deer ked organs was examined by immunofluorescence analysis. The relationship of *Bartonella* strains isolated from sika deer and arthropods were examined by a phylogenetic analysis based on concatenated sequences of the *gltA*, *rpoB*, *ftsZ*, and *ribC* genes, followed by a BLAST search for *gltA* and *rpoB*.

**Results:**

*Bartonella* prevalence in deer keds was 87.9% by real-time PCR and 51.5% in culture and that in the ticks was 8.3% by real-time PCR and 2.8% in culture. The mean number of *Bartonella* bacteria per ked was calculated to be 9.2 × 10^5^ cells. *Bartonella* aggregates were localized in the midgut of the keds. The phylogenetic analysis and BLAST search showed that both the host deer and the keds harbored two *Bartonella* species (lineages B and C), while *B. capreoli* (lineage A) was not detected in the keds. Two novel *Bartonella* species (lineages D and E) were isolated from one ked.

**Conclusions:**

*Lipoptena fortisetosa* likely serves as a vector of at least two *Bartonella* species (lineages B and C), whereas ticks do not seem to play a significant role in the transmission of *Bartonella* between sika deer based on the lower detection rates of *Bartonella* in ticks compared to keds. *Bartonella* species in lineages D and E appear to be *L. fortisetosa*-specific strains.
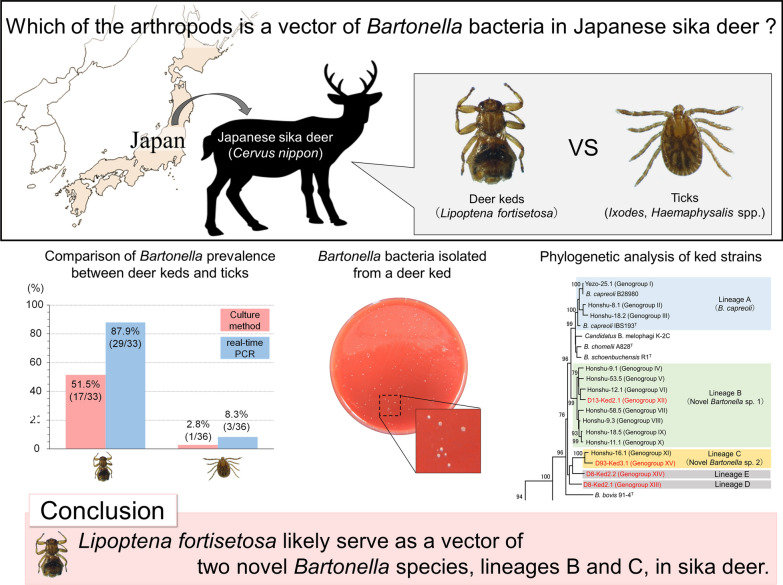

## Background

*Bartonella* species are Gram-negative, facultative intracellular bacteria. To date, more than 30 species and three subspecies have been described in the genus [1]. Several hematophagous arthropods are known to serve as vectors for the transmission of *Bartonella* bacteria to mammalian hosts, including sand fly (*Lutzomyia verrucarum*) for *B. bacilliformis* [2], human body louse (*Pediculus humanus humanus*) for *B. quintana* [3, 4] and cat flea (*Ctenocephalides felis*) for *B. henselae* [5]. Within the last two decades, it has been shown that *Lipoptena*, *Hippobosca* and *Melophagus* keds in the family Hippoboscidae are involved in the transmission of *Bartonella* bacteria to ruminants [6].

Deer keds (*Lipoptena* spp.) are recognized as obligate ectoparasites of ruminants but they can accidentally infest humans and other mammals [7, 8]. The keds have an atypical life-cycle compared with that of other hematophagous arthropods. After a winged ked attaches to its host’s body, it sheds its wings and then loses the ability to fly. Wingless keds are thought to remain on their initial hosts. One possible exception to this general rule is the movement of the Neotropical deer ked (*L. mazamae*) from a mother deer to its offspring due to close contact [9]. After blood-feeding on the host, a female ked lays a larva on the host body, and the larva pupates immediately. Although the pupa is thought to hatch in the summer or early autumn, precise information on the hatch-timing is lacking.

In Europe, *L. cervi* is the commonest deer ked found on red deer (*Cervus elaphus*), roe deer (*Capreolus capreolus*) and moose (*Alces alces*) [10]. *Bartonella*-derived DNA was first detected in wingless *L. cervi* collected from roe deer in France [11], with the high detection rate (93.8%) indicating that *L. cervi* may be epidemiologically associated with the transmission of *Bartonella* in this deer species. *Bartonella schoenbuchensis* was subsequently isolated from 73.3% of wingless *L. cervi* collected from roe deer and red deer in Germany [12]. Immunohistochemical analysis has also revealed the presence of large bacterial masses consisting of *B. schoenbuchensis* in the midguts of deer keds [12]. In addition, *B. schoenbuchensis* DNA was detected in wingless *L. cervi* (83.3%) collected from white-tailed deer in Massachusetts, USA [13], and *Bartonella* DNA was detected not only in developing larvae (71%) but also in winged *L. cervi* (6.4%) in Hungary [14]. In Finland, *B. schoenbuchensis*-like DNA was also detected from 13 pupae and one winged *L. cervi* collected in the field [10]. This epidemiological evidence raises the possibility that *B. schoenbuchensis* may be transstadially transmitted from the pupal to the winged stages of *L. cervi*.

The Japanese sika deer (*Cervus nippon*) is native to Japan and genetically divided into six subspecies, among which Honshu deer (*Cervus nippon centralis*) is widely distributed throughout the country, with the exception of Hokkaido, Shikoku and Kyushu islands. Yezo deer is also a subspecies (*Cervus nippon yesoensis*) of sika deer and inhabits only Hokkaido island [15]. In a previous study [16], we isolated *Bartonella* bacteria from 67.6% of Honshu deer and 50% of Yezo deer examined. A genetic analysis showed that Honshu and Yezo deer harbor at least three *Bartonella* species, including *B. capreoli* and two novel *Bartonella* species. Hematophagous arthropods, such as deer keds and ticks, were frequently detected on sika deer that tested positive for *Bartonella* bacteria. These observations suggest that these ectoparasites are involved in the transmission of *Bartonella* bacteria between sika deer. To date, there have been no studies investigating the role of hematophagous arthropods in the transmission of *Bartonella* bacteria between sika deer. The aim of the study reported here was to determine whether deer keds and/or ticks serve as vectors of *Bartonella* bacteria in sika deer. To achieve this aim, we used a variety of bacteriological, molecular biological and immunohistochemical techniques.

## Methods

### Sample collection

Between 2009 and 2012, 17 Honshu deer were captured in Nara (*N* = 8) and Shizuoka (*N* = 9) Prefectures, Japan. Blood samples were collected from the deer and immediately transferred into EDTA-containing collection tubes. The blood samples collected from Nara Prefecture were utilized for the isolation of *Bartonella* bacteria in our previous study [16]; the blood samples from Shizuoka Prefecture were used in the present study. The frozen blood samples were sent to the Laboratory of Veterinary Public Health, Department of Veterinary Medicine, College of Bioresource Sciences, Nihon University and stored at − 70 °C until examined.

A total of 38 wingless deer keds were collected from the deer captured in Nara (*N* = 10) and Shizuoka Prefectures (*N* = 28). All of the keds were morphologically identified as *Lipoptena fortisetosa*. Five deer keds from Shizuoka Prefecture were used for immunofluorescence analysis to determine the distribution of *Bartonella* bacteria within the bodies of the insects. A total of 36 ticks were collected from the deer in Nara (*N* = 33) and Shizuoka Prefectures (*N* = 3). Based on morphological analysis under stereomicroscopic observations these ticks were identified as *Haemaphysalis flava* (*N* = 16), *H. megaspinosa* (*N* = 15), *H. longicornis* (*N* = 4) and *Ixodes monospinosus* (*N* = 1). The live arthropod samples were immediately sent to the same laboratory under room temperature or refrigeration conditions (approx. 4 °C).

### Isolation of *Bartonella* bacteria from deer blood samples and arthropods

Isolation of *Bartonella* bacteria from deer blood samples was performed according to the procedure reported by Sato et al. [16]. To isolate *Bartonella* bacteria from the keds and ticks, first the surface of each arthropod was sterilized for 10 min with 500 μl of 70% ethanol containing 0.1% povidone-iodine, following which the arthropod was washed twice (1 min each wash) with 0.01 M phosphate buffered saline (PBS) containing 0.5% fetal bovine serum (FBS; Life Technologies, Carlsbad, CA, USA). Each arthropod was then homogenized in 400 μl of sodium sucrose glutamic acid buffer (10 mM sodium phosphate, 220 mM sucrose and 0.50 mM l-glutamic acid) for 1 min using a Micro Smash MS-100R homogenizing system (Tomy Seiko Co., Ltd., Tokyo, Japan) set at 3000 rpm, 4 °C. An aliquot (200 μl) of the homogenate was mixed with 200 μl of medium 199 supplemented with 1 mM sodium pyruvate solution and 20% volume of FBS, and 200 μl of this mixture was inoculated onto a heart infusion agar (HIA) plate (Difco, Becton Dickinson, Spark, MD, USA) containing 5% rabbit blood.

The inoculated plates were incubated at 35 °C in a moist atmosphere under 5% CO_2_ for up to 4 weeks. Bacterial colonies on HIA were tentatively identified as *Bartonella* based on colony morphology (small, gray or cream-yellow, round colonies), and five colonies per sample were subcultured on a fresh HIA plate using the same conditions as for the primary culture.

### *Bartonella* DNA detection by real-time PCR

A 200-µl aliquot of the homogenate from each arthropod was used for the extraction of DNA using InstaGene Matrix (Bio-Lad, Hercules, CA, USA). Real-time PCR targeting the 16S−23S internal transcribed spacer (ITS) region of *Bartonella* [17, 18] was used as a molecular screening for *Bartonella* DNA. To avoid false-negative reactions, TaqMan Exogenous Internal Positive Control (Exo IPC) Reagents (Applied Biosystems, Foster City, CA, USA) were also added to each well according to the manufacturer’s instructions. The reaction mixture (25 μl) consisted of 2.5 μl of the DNA extracts, 1.25 μl of each primer (10 μM), 12.5 μl of 2× TaqMan Fast PCR Master Mixture, 1.25 μl of FAM-labeled probe (5 μM), 2.5 μl of 10 × Exo IPC Mix including the specific primers and VIC-labeled probe, 0.5 μl of Exo IPC DNA (Applied Biosystems) and 3.25 μl of nuclease-free water. Three non-template controls (nuclease-free water) were prepared as a negative control for each PCR. Real-time PCR was performed under the following conditions: 50 °C for 2 min to activate uracil-N glycosylase; 95 °C for 10 min to activate the DNA polymerase; then 95 °C/15 s and 60 °C/1 min for 45 cycles. The targeted DNA was amplified using the 7500 Fast/Real-Time PCR system (Applied Biosystems), and fluorescence was detected through the FAM channel for ITS amplification and the VIC channel for IPC amplification.

### Estimation of the total number of *Bartonella* bacteria in keds by quantitative real-time PCR

We used quantitative real-time PCR (qRT-PCR) targeting the ITS of *Bartonella* species to estimate the total number of *Bartonella* bacteria in each culture-positive ked. A standard curve for estimating ITS copy number in each sample was constructed using serial dilutions of plasmid DNAs; the ITS amplicons from ked strains amplified by the 16SF and 23S1 primers [19] were inserted into a pGEM-T easy plasmid vector (Promega, Madison, WI, USA) and serially diluted from 1 × 10^1^ to 1 × 10^7^ copies/μl with nuclease-free water. As the *Bartonella* genome carries two ITS copies [20], the total number of *Bartonella* per ked was calculated by dividing the detected number of ITS copies by two copies.

### Immunofluorescence analysis to determine the distribution of *Bartonella* bacteria in deer keds

Immunofluorescent analysis was performed to investigate the distribution of *Bartonella* bacteria in deer keds. After fixation in 4% paraformaldehyde/PBS, the keds were sectioned toward the sagittal direction using a cryofracture technique and adhesive film [21]. *Bartonella* bacteria inside the ked body were visualized by indirect immunofluorescence staining. First, mouse anti-sera was made by immunizing mice with the four deer *Bartonella* strains (Honshu-8.1, Honshu-9.1, Honshu-9.3 and Yezo-25.1) [16] in our laboratory. Then, the anti-sera (1:100) diluted with 1% bovine serum albumin/PBS were applied to the sections and incubated for 2 h at room temperature; binding of the primary antibodies was detected using Alexa Fluor 488 conjugated goat anti-mouse IgG antibodies (Invitrogen, Carlsbad, CA, USA) for 1 h at room temperature. The actin fiber of the cytoskeleton of the deer ked was counterstained with Alexa Fluor 568 phalloidin (Invitrogen). Normal mouse serum was used as a negative control for each sample. The distribution of the *Bartonella* bacteria and actin fibers were visualized using a fluorescence microscope (model IX71) with the appropriate fluorescence filters (Olympus, Tokyo, Japan). Digital images of each section were captured for processing and analysis using the imaging software ‘cellSens’ (Olympus).

### Genogrouping of *Bartonella *strains based on the *gltA* and *rpoB* sequences

Genomic DNA was extracted from whole bacterial cells using InstaGene Matrix (Bio-Rad) and subjected to genus-specific PCR targeting the citrate synthase gene (*gltA*) [22] and RNA polymerase beta-subunit-encoding gene (*rpoB*) [23]. The PCR amplicons were purified using the Spin Column PCR product purification kit (Bio Basic Inc., Markham, ON, Canada) and sequenced directly using Applied Biosystems’ BigDye Terminator Cycle Sequencing Ready Reaction kit and Genetic Analyzer 3130. The obtained *gltA* and *rpoB* sequences were compared with those of the representative deer *Bartonella* strains from 11 genogroups [16] and genomic sequences of prokaryotes registered in the International Nucleotide Sequence Database (INSD) by BLAST search. Novel genogroup numbers were assigned to strains with new sequence variants of *gltA* and/or *rpoB*.

### Classification of *Bartonella* strain lineages based on phylogenetic analysis

The lineages of the genogroups were classified as described previously [16]. Thus, a representative strain of each novel genogroup was submitted to additional PCR and DNA sequencing analyses of the cell-division protein gene (*ftsZ*) [24] and the riboflavin synthase gene (*ribC*) [25]. A phylogenetic tree was constructed from the concatenated sequences of *gltA*, *rpoB*, *ftsZ*, and *ribC* using the maximum-likelihood method with the General Time Reversible model in MEGA 6 [26].

## Results

### Prevalence of *Bartonella* bacteria in sika deer and hematophagous arthropods

*Bartonella* bacteria were isolated from eight of the nine deer (88.9%) captured in Shizuoka Prefecture and subsequently isolated from 17 of 33 (51.5%) deer keds, whereas only one (*H. megaspinosa*) of 36 ticks (2.8%) was positive for *Bartonella* bacteria. *Bartonella* DNA was detected in 29 of 33 keds (87.9%), but only in three of 36 ticks (8.3%). The *Bartonella*-positive ticks were identified as *H. megaspinosa* (*N* = 1) and *H. longicornis* (*N* = 2). IPC amplification was confirmed in all of the reactions.

### *Bartonella *cell counts by qRT-PCR and the distribution of *Bartonella* bacteria within deer keds

In the 17 keds from which *Bartonella* bacteria were isolated (see above), a qRT-PCR analysis indicated that the total number of *Bartonella* bacteria per ked ranged from 1.4 × 10^4^ to 7.0 × 10^6^ cells, with a mean of 9.2 × 10^5^ per ked (Table 1).

**Table 1:** Cell counts of *Bartonella* bacteria in deer keds using *Bartonella*-specific quantitative real-time PCR

Deer ked ID number	Number of *Bartonella* bacteria per ked
D8-Ked1	2.7 × 10^5^
D8-Ked2	1.4 × 10^4^
D8-Ked3	2.1 × 10^5^
D8-Ked4	1.6 × 10^4^
D8-Ked5	3.2 × 10^4^
D12-Ked1	8.9 × 10^4^
D13-Ked1	1.5 × 10^5^
D13-Ked2	1.1 × 10^5^
D13-Ked3	6.1 × 10^4^
D89-Ked2	3.4 × 10^5^
D91-Ked1	2.1 × 10^5^
D91-Ked5	2.3 × 10^6^
D93-Ked1	3.1 × 10^6^
D93-Ked2	2.2 × 10^5^
D93-Ked3	1.7 × 10^5^
D93-Ked4	7.0 × 10^6^
D96-Ked2	1.5 × 10^6^
Mean number per ked (± standard error)	9.2 × 10^5^ (± 4.2 × 10^5^)

**Figure 1:**
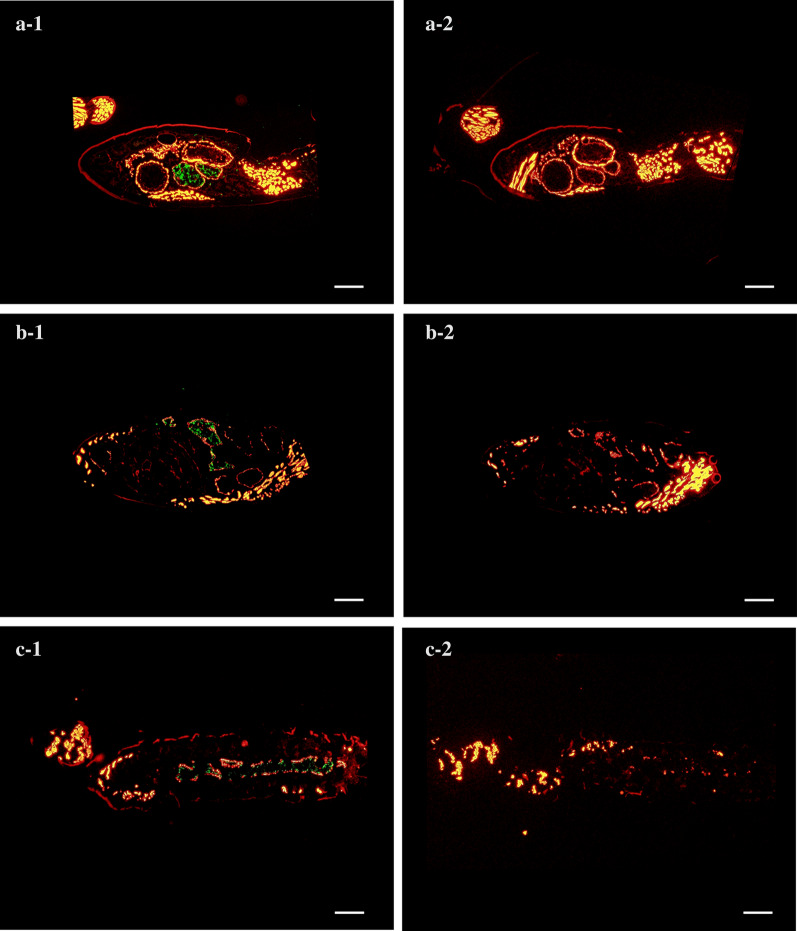
Distribution of *Bartonella* bacteria inside wingless deer keds (*Lipoptena fortisetosa*).** a**,** b**,** c** Sagittal sections of three different deer keds, respectively. **a-1**, **b-1**, **c-1 **Immunostained sagittal sections of the keds using anti-sika deer strains mouse sera and Alexa Fluor 488 conjugated goat anti-mouse IgG antibodies. **a-2**, **b-2**, **c-2** Sagittal sections of the keds treated with normal mouse serum as a negative control. Actin fibers in the deer keds were counterstained with Alexa Fluor 568 phalloidin in order to aid determination of *Bartonella* distribution. Scale bar: 100 µm.

**Figure 2:**
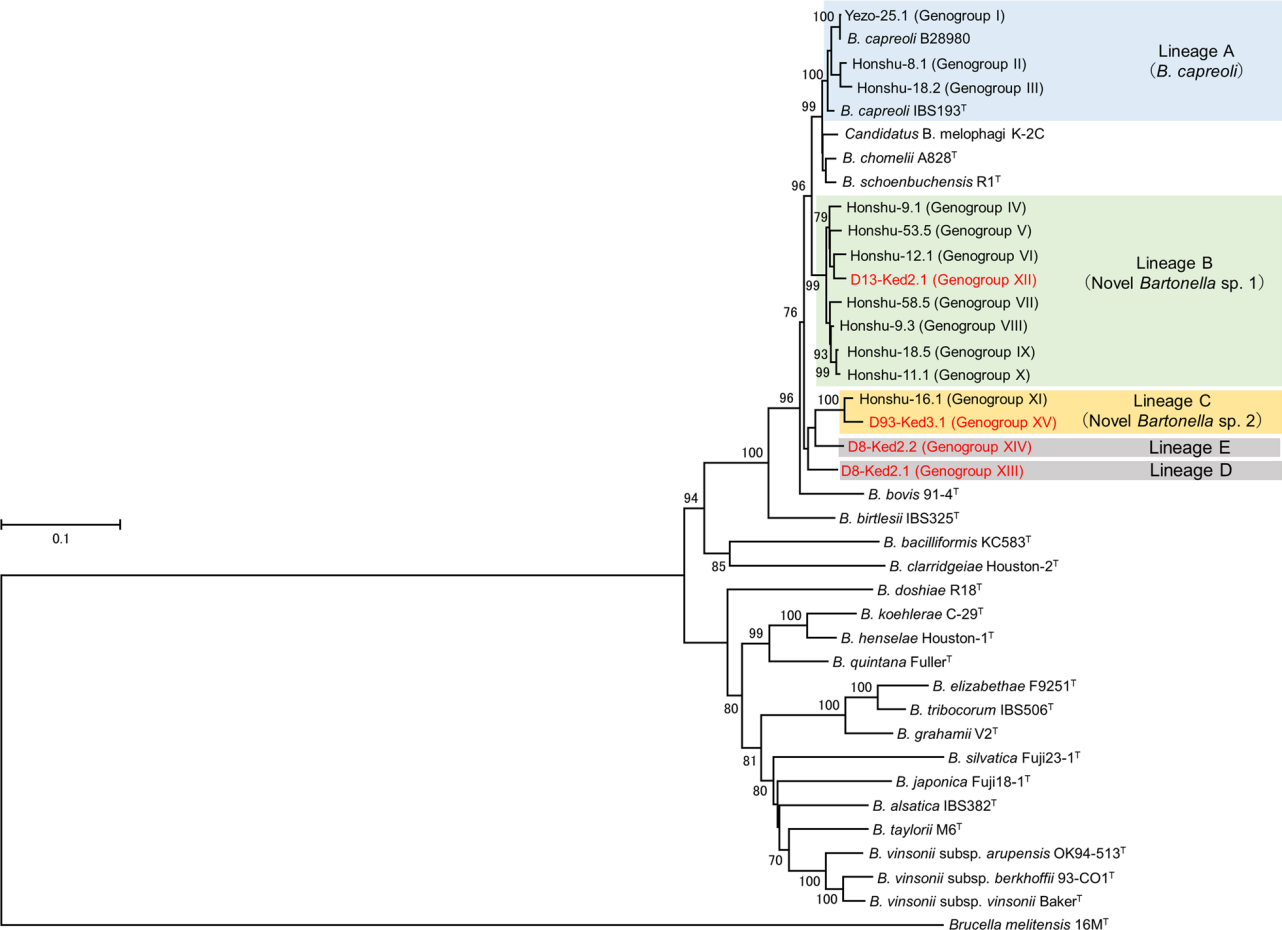
Phylogenetic tree of *Bartonella* strains from deer ked and Japanese sika deer and other ruminants. The tree was produced using the maximum-likelihood method based on the General Time Reversible model. A discrete gamma distribution was used to model evolutionary rate differences among sites (5 categories [G, parameter = 0.4518]). Four representative strains (D8-Ked2.1, D8-Ked2.2, D13-Ked2.1 and D93-Ked3.1) from deer keds, 11 representative strains (Honshu-8.1, -9.1, -9.3, -11.1, -12.1, -16.1, -18.2, -18.5, -53.5, -58.5 and Yezo-25.1) from Japanese sika deer, the ruminant-associated *Bartonella* species (*B. bovis* 91-4^T^, *B. capreoli* IBS193^T^, *B. capreoli* B28980, *B. chomelii* A828^T^, *B. schoenbuchensis* R1^T^ and *candidatus* B. melophagi K-2C) and several other known *Bartonella* species were included in the tree. The tree was rooted using *Brucella melitensis* strain 16M^T^ as an outgroup. Bootstrap values with > 70% confidence are indicated at the tree nodes. Branch lengths are measured as the number of substitutions per site.

Examination of immunostained ked tissue sections showed that specific fluorescence indicating *Bartonella* aggregates was only present in the midguts of three keds (Fig. 1a-1, b-1, c-1). No fluorescence were observed in other organs of these keds with normal mouse sera (Fig. 1a-2, b-2, c-2).

### Genogrouping of *Bartonella* strains

A total of 120 *Bartonella* isolates (5 isolates per one sample) were recovered from 15 keds, one tick, and eight deer. More three isolates were obtained from two keds (ID# D91-Ked1 and D91-Ked5) because a few colonies of *Bartonella* bacteria were grown on the agar plates. As a result, a total of 123 isolates were obtained in the present study and were classified into 15 genogroups (I to XV); the genogroups XII (10 strains), XIII (three strains), XIV (two strains), and XV (10 strains) were newly found in the present study. The new sequence variants of the *gltA*, *rpoB*, *ftsZ*, and *ribC* in the representative strains from novel genogroups have been registered at INSD and accession numbers assigned to each of the variant sequences (Table 2).

**Table 2:** Accession numbers of genes of representative *Bartonella* strains from four novel genogroups

Novel genogroups	Representative strains	Accession numbers			
		*gltA*	*rpoB*	*ftsZ*	*ribC*
XII	D13-Ked2.1	LC485114	LC485118	LC485122	LC485126
XIII	D8-Ked2.1	LC485115	LC485119	LC485123	LC485127
XIV	D8-Ked2.2	LC485116	LC485120	LC485124	LC485128
XV	D93-Ked3.1	LC485117	LC485121	LC485125	LC485129

*gltA*, Citrate synthase gene; *rpoB*, RNA polymerase beta-subunit-encoding gene; *ftsZ*, cell-division protein gene; *ribC*, riboflavin synthase gene

### Lineage classification and sequence homology analysis of novel *Bartonella* genogroups

A phylogenetic analysis of representative strains of the 15 genogroups based on the concatenated sequences of the four housekeeping genes (Fig. 2) revealed that the genogroups were divided into five lineages (A, B, C, D and E) (Fig. 2). None of the ked strains belonged to lineage A. Ked strain D13-Ked2.1 (XII) was grouped in lineage B along with the seven deer strains consisting of Honshu-9.1 (IV) to Honshu-11.1 (X). Ked strain D93-Ked3.1 (XV) belonged to lineage C along with deer strain Honshu-16.1 (XI). Ked strains D8-Ked2.1 (XIII) and D8-Ked2.2 (XIV) formed monophyletic clades as lineages D and E, respectively.

The *gltA* and *rpoB* sequences of representative strains from the novel genogroups were compared with those of the bacterial genomic sequences registered in INSD using BLAST search (Table 3). The *gltA* and *rpoB* sequences of ked strain D13-Ked2.1 (XII) were identical to those of deer strains Honshu-9.1 and Honshu-18.5. The *rpoB* sequence of ked strain D13-Ked2.1 (XII) showed 98.8% similarity with that of deer strain Honshu-12.1. The *gltA* sequence of ked strain D8-Ked2.1 (XIII) showed 97.6% similarity with strain MUD detected from *Lipoptena* sp. in the USA. The *rpoB* sequence of ked strain D8-Ked2.1 (XIII) showed 99.6% similarity with five uncultured *Bartonella* strains from *L. cervi* in Poland. The *gltA* sequence of ked strain D8-Ked2.2 (XIV) showed 96.7% similarity with deer strains Honshu-9.1 and Honshu-18.5 from sika deer, whereas the *rpoB* sequence showed 97.2% similarity with an uncultured *Bartonella* strain detected from a white-tailed deer (*Odocoileus virginianus*) in the USA. The *gltA* and *rpoB* sequences of ked strain D93-Ked3.1 (XV) showed sequence similarity of 100% and 99.4% for *gltA* and *rpoB*, respectively, to deer strain Honshu-16.1.

**Table 3:** Novel genogroup classification based on a BLAST analysis of the *gltA* and *rpoB* genes

Representative strains (Genogroups)	*gltA* (338 bp)			*rpoB* (825 bp)		
	Strain (accession no.)	Scientific name of host	Similarity (%)	Strain (accession no.)	Scientific name of host	Similarity (%)
D13-Ked2.1 (XII)	Honshu-9.1 (AB703125) Honshu-18.5 (AB703129)	*Cervus nippon*	100	Honshu-12.1 (AB703146)	*Cervus nippon*	98.8
D8-Ked2.1 (XIII)	MUD (JX416234)	*Lipoptena* sp.^a^	97.6	Uncultured five *Bartonella* strains (MF580657–MF580661)^b^	*Lipoptena cervi*^c^	99.6
D8-Ked2.2 (XIV)	Honshu-9.1 (AB703125) Honshu-18.5 (AB703129)	*Cervus nippon*	96.7	Uncultured *Bartonella* species (AY805112)	*Odocoileus virginianus*^d^	97.2
D93-Ked3.1 (XV)	Honshu-16.1 (AB703131)	*Cervus nippon*	100	Honshu-16.1 (AB703149)	*Cervus nippon*	99.4

^a^*Lipoptena* sp. collected in the USA

^b^Strains BLC59KG, BLC63KG, BLC73KG, BLC107KG, and BLC202KG from deer keds in Poland

^c^*Lipoptena cervi* collected in Poland

^d^*Odocoileus virginianus* (White-tailed deer) captured in the USA

### Relationship of *Bartonella* lineages among hematophagous arthropods and sika deer

Fourteen deer keds and one tick harbored *Bartonella* strains of lineage B, whereas two keds (ID# D93-Ked3 and D96-Ked2) harbored lineage C strains (Table 4). Another ked, D8-Ked2, harbored two *Bartonella* strains classified in lineages D and E. Five of eight deer harbored *Bartonella* strains classified in lineage B, and the others harbored *Bartonella* strains classified in lineage A.

**Table 4:** Relationship of *Bartonella* lineages between hematophagous arthropods and sika deer

Hematophagous arthropod ID number	*Bartonella* lineage^a^		Deer ID number
	Arthropod strain	Deer strain	
D8-Ked1, D8-Ked3 D8-Ked4, D8-Ked5	B	A	Deer 8
D8-Ked2	D and E		
D11-Tick12	B	B	Deer 11
D12-Ked1	B	B	Deer 12
D13-Ked1, D13-Ked2, D13-Ked3	B	A	Deer 13
D89-Ked2	B	B	Deer 89
D91-Ked1, D91-Ked5	B	A	Deer 91
D93-Ked1, D93-Ked2, D93-Ked4	B	B	Deer 93
D93-Ked3	C		
D96-Ked2	C	B	Deer 96

^a^Lineage A represents *Bartonella capreoli* and lineages B to E are novel *Bartonella* species

Deer 11, 12, 89 and 93 harbored *Bartonella* strains of the same lineage (B) as the hematophagous arthropods D11-Tick12, D12-Ked1, D89-Ked2, D93-Ked1, D93-Ked2 and D93-Ked4. In contrast, deer 8, 13, 91 and 96 harbored *Bartonella* strains of lineage A or B, but no strains of the same lineages were detected from the keds that infested the deer.

## Discussion

In the present study, wingless keds collected from Japanese sika deer were morphologically identified as *Lipoptena fortisetosa* and found to harbor *Bartonella* bacteria at a high rate (87.9%). Halos et al. [11] first demonstrated that *Bartonella* bacteria were present at a high rate (93.8%) in wingless *L. cervi* collected from French roe deer. Since then, it has been reported that *Bartonella* bacteria are prevalent also in the keds collected from red deer and roe deer in Hungary [14] and Poland [27] and from moose in Norway [28]. Likewise, *Bartonella* DNAs were detected from both wingless *L. cervi* (83.3%) [13] and wingless *L. mazamae* (28.9%) collected from white-tailed deer in the USA [29], and from 100% of wingless *L. mazamae* collected from gray brocket deer in Brazil [30]. These epidemiological data show that *Lipoptena* keds harbor high levels of *Bartonella* bacteria and are likely to play an important role in transmitting the bacteria between various deer species.

We isolated *Bartonella* bacteria from 51.5% of the wingless *L. fortisetosa* collected. More than 1000 *Bartonella* colonies were recovered from a ked (ID# D93-Ked4) using the culture method, and a very high number of *Bartonella* cell counts (7.0 × 10^6^) were estimated by *Bartonella*-specific qRT-PCR. These data suggest that *L. fortisetosa* offer an optimum environment for *Bartonella* bacteria. Results from a survey of deer in Germany demonstrated that a large number of *Bartonella* colonies (> 1000 per *L. cervi*) could be recovered from *L. cervi* by the culture method, with the immunohistochemical and transmission electron microscopic analyses also showing that bacterial aggregates were only present in the ked midguts, suggesting that *Bartonella* bacteria proliferated in the midgut of *L. cervi* [12]. In the present study, our immunofluorescent analysis also found that *Bartonella* aggregates were only detected in the midgut of *L. fortisetosa*. This result is consistent with those of the previous study [12] and suggests that *Bartonella* bacteria may also propagate in the midgut of *L. fortisetosa*.

Although *Bartonella* DNAs were detected from *Ixodes ricinus* ticks in France [31] and Poland [32], it is unknown whether the *Bartonella* DNAs were host-borne or not. Moreover, it is suggested that the mere presence of *Bartonella* DNA in ticks is not enough to prove vector competence of *Bartonella* bacteria [33]. Tijsse-Klasen et al. [34] reported that *Bartonella* DNA was not found in 1719 questing *I. ricinus* nymph and adult ticks collected in the field in the Netherlands. In the present study, the isolation rate from ticks was considerably lower than that from keds, as *Bartonella* bacteria were isolated only from one engorged *Haemaphysalis* tick collected on a bacteremic sika deer. The lineage of the *Bartonella* isolated from tick D11-Tick12 was the same (lineage B) as that of the deer from which the tick was collected. This suggests that the *Bartonella* bacteria might have been present in the deer blood ingested by the tick. Therefore, it is possible that *Ixodes* and *Haemaphysalis* ticks do not play a significant role in the transmission of *Bartonella* in deer.

The phylogenetic analysis shows that novel genogroups XII and XV were included in lineages B and C, respectively. A BLAST search also indicated that ked strain D13-Ked2.1 (genogroup XII) was closest to deer strains Honshu-9.1, Honshu-18.5 and Honshu-12.1 in lineage B, whereas ked strain D93-Ked3.1 (genogroup XV) was closest to deer strain Honshu-16.1 in lineage C. In our previous study [16], the *Bartonella* strains in lineage A were identified as *B. capreoli*; however, the strains in lineages B and C did not cluster with known *Bartonella* species, suggesting that these strains are novel *Bartonella* species. From these results, genogroup XII in lineage B and genogroup XV in lineage C are also suggested to be novel *Bartonella* species. The ked strains D8-Ked2.1 (genogroup XIII) and D8-Ked2.2 (genogroup XIV) formed the new independent lineages D and E, respectively. Furthermore, the *gltA* and *rpoB* sequences of ked strain D8-Ked2.1 (genogroup XIII) were closest to those of uncultured *Bartonella* species detected from *Lipoptena* keds in the USA and Poland. Although the *gltA* sequence of ked strain D8-Ked2.2 (genogroup XIV) was similar to deer strains Honshu-9.1 and Honshu-18.5, the *rpoB* sequence of the same genogroup was similar to uncultured *Bartonella* species detected from white-tailed deer in the USA [35]. These findings support the suggestion that genogroup XIII in lineage D and genogroup XIV in lineage E may also be novel *Bartonella* species.

In our previous study, the deer *Bartonella* strains from Hokkaido, Wakayama and Nara Prefectures were classified in lineages A to C [16]. In the present study, the *Bartonella* strains from the deer in Shizuoka Prefecture were classified in lineages A and B, while most of the strains from *L. fortisetosa* were classified in lineages B and C. These data indicate that *L. fortisetosa* harbors two novel *Bartonella* species, namely lineages B and C, and may transmit the bacteria to Japanese sika deer. The other novel *Bartonella* species, namely lineages D and E, were found in only one ked (D8-Ked2), but not in sika deer or other deer species in other countries. Further analyses are required to determine whether these new lineages are deer ked-specific *Bartonella* species.

Deer keds are generally thought to stay on the same deer body after dropping their wings. However, Samuel and Trainer [9] found evidence that wingless *L. mazamae* on white-tailed deer can mechanically transfer between individuals during direct contact between deer. In the present study, the *Bartonella* lineages of deer keds differed from those of four sampled deer (deer 8, 13, 91, and 96), raising the possibility that wingless *L. fortisetosa* may also be able to transfer between individuals within the deer population. Given that *Bartonella* bacteria propagate in the ked midgut, *Lipoptena* keds may serve as a biological vector for transmitting *Bartonella* bacteria to deer.

It has been suggested that *B. schoenbuchensis* can be transmitted not only vertically from adult *L. cervi* to offspring [14], but also transstadially to the next growth stage of *L. cervi* [10]. Unfortunately, we did not obtain any pupae and winged adult keds and so could not examine these possibilities in our study. Further studies are needed to resolve the questions of transovarial and transstadial transmission of *Bartonella* bacteria in *L. fortisetosa*.

Interestingly, *B. capreoli* (lineage A) was not found in any deer keds examined in our study, although this *Bartonella* species was isolated from three sika deer. To date, *B. capreoli* has not been detected from *L. cervi* and/or *L. mazamae* in Europe and the USA although this species has frequently been isolated from several deer species. The absence of this *Bartonella* species in *Lipoptena* species suggests that other hematophagous arthropods may be involved in the transmission of *B. capreoli* between deer populations.

## Conclusions

Our data indicate that *L. fortisetosa* likely serves as a vector of at least two *Bartonella* species in Japanese sika deer, similar to *L. cervi* and *L .mazamae* transmitting *Bartonella* bacteria to deer in Europe and the USA. In contrast, *Ixodes* and *Haemaphysalis* ticks may not play a significant role in the transmission of *Bartonella* in the sika deer.

## Data Availability

The nucleotide sequences of the ked strains were submitted to INSD under accession numbers LC485114–LC485129.
